# Optical microscopic imaging, manipulation, and analysis methods for morphogenesis research

**DOI:** 10.1093/jmicro/dfad059

**Published:** 2023-12-15

**Authors:** Takanobu A Katoh, Yohsuke T Fukai, Tomoki Ishibashi

**Affiliations:** Department of Cell Biology, Graduate School of Medicine, The University of Tokyo, 7-3-1 Hongo, Bunkyo-ku, Tokyo 113-0033, Japan; Nonequilibrium Physics of Living Matter RIKEN Hakubi Research Team, RIKEN Center for Biosystems Dynamics Research, 2-2-3 Minatojima-minamimachi, Chuo-ku, Kobe, Hyogo 650-0047, Japan; Laboratory for Physical Biology, RIKEN Center for Biosystems Dynamics Research, 2-2-3 Minatojima-minamimachi, Chuo-ku, Kobe, Hyogo 650-0047, Japan

**Keywords:** optical microscopy, segmentation tool, tracking tool, morphogenesis, mechanobiology

## Abstract

Morphogenesis is a developmental process of organisms being shaped through complex and cooperative cellular movements. To understand the interplay between genetic programs and the resulting multicellular morphogenesis, it is essential to characterize the morphologies and dynamics at the single-cell level and to understand how physical forces serve as both signaling components and driving forces of tissue deformations. In recent years, advances in microscopy techniques have led to improvements in imaging speed, resolution and depth. Concurrently, the development of various software packages has supported large-scale, analyses of challenging images at the single-cell resolution. While these tools have enhanced our ability to examine dynamics of cells and mechanical processes during morphogenesis, their effective integration requires specialized expertise. With this background, this review provides a practical overview of those techniques. First, we introduce microscopic techniques for multicellular imaging and image analysis software tools with a focus on cell segmentation and tracking. Second, we provide an overview of cutting-edge techniques for mechanical manipulation of cells and tissues. Finally, we introduce recent findings on morphogenetic mechanisms and mechanosensations that have been achieved by effectively combining microscopy, image analysis tools and mechanical manipulation techniques.

## Introduction

The formation of ordered multicellular structures during embryonic development has long been a significant subject of research. Early studies attempted to elucidate the morphogenetic mechanisms during development through histological observations [1]. In the post-Genome Project era, major progress has been made in uncovering the genetic and signaling pathways that govern the morphogenesis of diverse yet robust organ and tissue structures.

Morphogenesis is driven by entities that are interlaced with gene regulatory networks, specifically, the cell shape alterations and movements that are propelled by mechanical forces [2]. To understand how genetic programs orchestrate the formation of structures and trace the causal relationships behind the process, we must both characterize the morphologies and dynamics at the single-cell scale and comprehend how physical forces serve as signaling components and drivers of deformation.

The field has seen the development of sophisticated microscopy techniques that improve observation speed, resolution and imaging depth. Various image analysis techniques have also been developed to support large-scale, single-cell-level analyses. Particularly, the development of machine-learning methods has enabled the quantification of challenging images [3,4]. Efforts have also been made to make those techniques widely accessible. Furthermore, advancements in imaging, manipulation and analysis techniques have facilitated the comprehensive examinations of mechanical processes during morphogenesis [5–9]. Specifically, the integration of diverse technologies has revealed intricate interactions between shapes, forces and signaling pathways [10,11].

Despite the importance of combining different techniques, this growing sophistication demands increased expertise. With this background, this review aims to provide an overview of these techniques and briefly introduce them for practical applications. First, we introduce microscopies for multicellular imaging. Next, we review image analysis techniques and software tools, focusing on cell instance segmentation and tracking which are essential for uncovering the relationships between microscopic cellular properties and macroscopic morphogenetic dynamics. Then we introduce cutting-edge mechanical manipulation techniques to dissect mechanical processes in living systems. Finally, we discuss the latest research findings morphogenesis and illustrate how our understanding has progressed through the use of these imaging and analysis techniques.

## Three-dimensional imaging microscopy for morphogenesis research

Cell morphology and movement bridge the macroscopic morphology of organs and tissues and their underlying microscopic molecular mechanisms. Advancements in imaging techniques have substantially contributed to morphogenesis studies by dissecting multicellular dynamics during development [10,11]. Although requirements for factors such as area, speed, resolution and phototoxicity often conflict with each other [11], the development of innovative microscopes in recent decades has enabled the expansion of the limits.

A commonly used microscopy in this field is scanning laser confocal microscopy. Unlike wide-field microscopy, confocal microscopy achieves the distinction of *z*-planes by eliminating light originating from regions outside the focal plane using a pinhole, thus enabling the acquisition of three-dimensional (3D) images. Notably, with recent advancements in detector sensitivity (e.g. the development of GaAsP detectors), confocal microscopy has facilitated the acquisition of clearer images. Additionally, the integration of image scanning microscopy [12] principles into confocal microscopy, for example, the use of array detectors such as AiryScan (Zeiss) and NSPARC (Nikon) has significantly enhanced resolution and achieving sub-diffraction limit resolution.

However, answering biological questions in morphogenesis sometimes requires faster, wider and deeper image acquisition beyond the capability of scanning confocal microscopy. Here we focus on three major microscopy techniques that have various advantages to scanning confocal microscopy in some cases, with emphasis on recent technical advancements. First, we introduce spinning-disk confocal microscopy that empowers live 3D imaging through rapid image acquisition capabilities. Then we describe two-photon microscopy that excels at imaging deeper regions within the tissue. Finally, we highlight the light-sheet microscopy that is characterized by its rapid image acquisition, expansive field of view, and considerable depth penetration, and capable of capturing entire tissue images. The typical specifications for those microscopies are summarized in Table 1.

**Table 1.** Typical features of microscopy techniques

	Microscopy							
	Conventional confocal		Spinning-disk Confocal		Two-photon		Light-sheet	
Objective	4×/0.16	100×/1.45	4×/0.16	100×/1.45	10×/0.6 Obj	25×/1.0 Obj	5×/0.1&5×/0.16	10×/0.2&20×/1.0
Speed	<∼30 fps		<∼1000 fps		<∼30 fps		<∼100 fps	
Resolution (*x *× *z*)	∼1.5 × 40 µm	∼0.2 × 1 µm	2 × 100 µm	∼0.2 × 1 µm	>0.7 × 7 µm	>0.4 × 2.5 µm	∼2 × 14 µm∼	∼0.3 × 2 µm
Field	∼4500 µm	∼180 µm	∼5500 µm	∼220 µm	∼1800 µm	∼720 µm	∼3500 µm	∼1000 µm
Imaging depth	<100 µm		<100 µm		<1000 µm [19]		<100 µm	

### Spinning-disk confocal microscopy

**Fig. 1.**
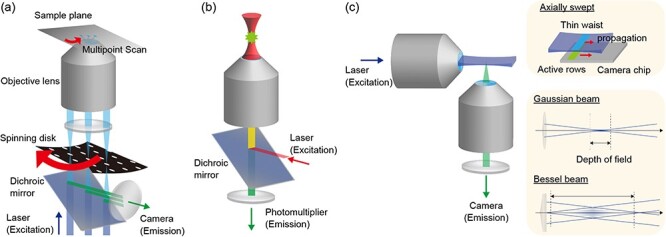
**Comparison of microscopy** (a) Schematic of spinning-disk confocal microscopy. A rotating spinning-disk (illustrated in black) with numerous pinholes is placed in the equivalent sample plane, which scans the sample plane with multiple points (blue lines). (b) Schematic of two-photon excitation microscopy. The excitation laser, illustrated in red, is focused in the sample and excites the fluorophore when two photons are simultaneously absorbed by fluorophores. The emitted fluorescence, illustrated in green, has a shorter wavelength than that of the excited laser. (c) Schematic of the light-sheet microscopy. A sample (not illustrated), usually embedded in gel, is located at the intersecting point of the optical axes of two objective lenses. One objective illuminates a single plane in the sample (illustrated in blue plane), and another objective lens observes this plane in a single shot. The upper right panel shows a schematic of axially swept light-sheet microscopy. The lower right panel shows the comparison of the Gaussian beam and the Bessel beam. The blue line represents the outline of light rays, and the light blue regions in the Bessel beam represent the side lobes.

Conventional confocal microscopy has been used as a standard method for acquiring 3D images. However, the speed and phototoxicity are among the problems for live 3D imaging due to the image acquisition process that requires repeated laser scanning. Spinning-disk confocal microscopy emerged as an alternative to address these limitations. In contrast to standard point-scanning methods, which illuminate one point in a sample at a time and then scan the specimen, spinning-disk confocal microscopes illuminate multiple (∼1000) points simultaneously using a pinhole array disk (Table 1, Fig. 1a). This reduces the time required for image acquisition and laser scanning. The system can be extended to simultaneous two-color live 3D imaging, making it suitable for applications such as calcium imaging [13]. Most spinning-disk confocal microscopes are installed at camera ports, enabling the construction of a custom-built optical pathway, such as a pathway for optical stimulation or optical tweezers, using an episcopic illuminator port [14,15].

Recently, advanced microscopy methods have been developed by combining spinning-disk confocal microscopy with other techniques, such as two-photon microscopy (described further) or super-resolution microscopy techniques, yielding better performance in terms of imaging depth and resolution. For example, two-photon microscopy with the improved pinhole disk-equipped spinning-disk confocal microscopy achieved observation of ∼100 µm deep regions [16]. In addition, structured illumination microscopy (SIM) was combined with spinning-disk confocal microscopy by using a disk with a fine stripe pattern [17]. This microscope improved the resolution and achieved ∼120 nm *x–y* resolution with a speed of 30–100 frames/s.

Compared with point-scanning confocal microscopy, spinning-disk microscopy has the disadvantage of a relatively lower resolution along the *z*-axis. This is because the fixed pinhole size makes it impossible to match the size to the optimal one—one Airy Unit (AU) of the system. Generally speaking, spinning-disk microscopes are designed for high-magnification objective lenses; for example, the 1 AU size of 100×/1.35NA objective is ∼50 µm, which matches the typical spinning-disk pinhole size.

### Two-photon microscopy

Two-photon excitation microscopy is suitable for deep-tissue observation (Table 1, Fig. 1b). Unlike the majority of fluorescent microscopes that rely on the absorption of a single photon to excite fluorophores, this technique uses two-photon absorption. Two-photon excitation refers to the excitation of a single fluorophore by simultaneous absorption of two or more photons with lower energy than that of the fluorescent light. In contrast to single-photon excitation, for which the excitation light needs to have a shorter wavelength (higher energy) than that of the fluorescence, the wavelength of the excitation light is longer than that of the fluorescence.

Because of the low probability of two-photon absorption, the excitation light needs to have a high peak flux. Therefore, infrared femtosecond pulsed lasers are typically used as the excitation light. At infrared wavelengths, typical biological specimens have a spectral range called an optical window characterized by low absorption and scattering [18], making them suitable for deep-tissue observation, in the best case, a 1 mm or deeper region can be observed [19]. Because of the long wavelength of the excitation laser, the resolution of two-photon microscopy is slightly lower than that of conventional confocal microscopy (see Table 1).

### Light-sheet microscopy

For 3D observations using a low-magnification objective lens, light-sheet microscopy is likely a better option (Table 1, Fig. 1c) [20,21]. In the context of resolution and speed, light-sheet microscopy outperforms confocal microscopy when conducting observations at magnifications typically set at 20× or lower (see Table 1). The assembly of a light-sheet microscope differs from that of a conventional microscope. Except for single-objective light-sheet microscopes [22], most light-sheet microscopes have two (or more) objective lenses arranged orthogonally for illumination and observation. By segregating the illumination and observation pathways, light-sheet microscopy presents three distinct advantages: reduced phototoxicity, enhanced imaging speed and improved *z*-resolution.

From the perspective of phototoxicity and photobleaching, light-sheet microscopy exhibits a distinct advantage by employing the illumination objective to selectively irradiate excitation light solely within the desired plane, whereas confocal microscopy indiscriminately illuminates excitation light in both the upper and lower regions of the focal point. In terms of imaging speed, the light-sheet microscope possesses the capability to capture an image in a single acquisition without the need for *xy*-scanning, as required in conventional confocal microscopy. As a result, it can rapidly acquire 3D images by scanning the sample or the light sheet along the *z*-axis achieved by moving the focal plane of the objective lens at high speed [23,24]. For example, the light-sheet microscope can take a 3D movie of a moving *Amoeba* with a size of hundreds of micrometers [24]. The last advantage pertains to *z*-resolution. In light-sheet microscopy, the *z*-resolution demonstrates a linear correlation with the numerical aperture (NA) of the illumination light. In contrast, the *z*-resolution of confocal microscopy is contingent upon NA^2^. Consequently, when using a lower-magnification objective lens, typically characterized by lower NA values, light-sheet microscopy attains superior *z*-resolution in comparison to confocal microscopy (Table 1).

In light-sheet microscopy, a challenge arises from the trade-off between the field of view and *z*-resolution. The *z*-resolution in light-sheet microscopy is governed by the beam waist of the light sheet, and consequently, a thinner light sheet yields higher *z*-resolution. In turn, the width (or depth) of the light sheet is directly correlated with the field of view. In the case of Gaussian beams, the width (or depth) of the light sheet is directly correlated to the Rayleigh length, which is defined as πω^2^/λ, where ω and λ represent the beam waist and the wavelength, respectively. This equation implies that as the *z*-resolution is increased, ω decreases, consequently reducing the Rayleigh length. As a result, *z*-resolution and field of view are conflicting factors. Nonetheless, recent innovations in light-sheet microscopy have effectively extended the constraints of this limitation, primarily achieved through the creation of a larger field of view by segmenting narrow areas or leveraging the diffraction properties of light.

One effective method is axially swept light-sheet microscopy (Fig. 1c) [21]. In this technique, the in-focus region of the light sheet is dynamically swept across the sample through the utilization of a remote focus system. Consequently, this specialized light sheet effectively ‘scans’ the sample by employing only the thinner portion of the Gaussian beam (the region near the beam waist). Synchronization of the active pixels of the complementary metal-oxide semiconductor (CMOS) camera with this thinner portion of the light sheet allows for the exclusion of blur. This synchronization ensures that only images captured within the thin sheet region are recorded, achieved by sweeping a narrow strip of active pixels across the sensor in this type of camera while maintaining acquisition speed.

An alternative approach involves the use of a light sheet generated by non-Gaussian beams, such as Bessel beams (Fig. 1c) and lattice light-sheet microscopy (LLSM). In Bessel-beam light-sheet microscopy [25,26], the light sheet is created using a Bessel beam, which is constructed from an oriented ring-shaped planar wave, often facilitated by axicon lenses. The intensity profile of the cross-section of the Bessel beam exhibits a single bright region with a smaller diameter that is narrower than the diffraction limit (referred to as the zero-order Bessel beam), along with numerous side lobes arranged in concentric circles. This zero-order Bessel beam extends deeply along the optical axis, resulting in Bessel beam light-sheet microscopy possessing a wide field of view with a thin illumination light sheet.

One of the most cutting-edge advancements in light-sheet microscopy is LLSM [27]. LLSM employs a spatial light modulator to illuminate the sample with a spatially modulated *optical lattice* light sheet. LLSM demonstrates a noteworthy reduction in sidelobes, the higher-order diffraction waves responsible for blurring, in comparison to Bessel beam light-sheet microscopy. However, the construction of LLSM necessitates more intricate and expensive optical components, including a spatial light modulator. Remarkably, this light sheet represents one of the thinnest reported to date and extends deeply along the optical axis. Furthermore, this lattice-like structure serves as structured illumination for SIM. This mechanism overcomes the diffraction limit of conventional light-sheet microscopy; typically, LLSM achieves ∼150 × 280 nm in *x–z* resolution. Moreover, the application of adaptive optics to LLSM that corrects sample-induced aberrations by a deformable mirror, Liu *et al.* succeeded in observing deep tissue regions exceeding 100 µm in depth [28].

## Image analysis tools for multicellular systems—cell segmentation, tracking and more

Advances in the microscopy techniques and increases in computational resources have enhanced the quality and quantity of data obtainable from imaging. For example, whole-embryo live imaging is possible for various model organisms [11,29], enabling the observation of cellular shapes, motion and differentiation. Accordingly, computational methods for quantification have been actively developed, paving the way for quantitative understanding of multicellular phenomena.

As partly discussed in the following sections, characterizing single-cell scale properties provides opportunities to understand the relationship between cellular properties and macroscopic morphologies [30–32], attribute macroscopic deformations to cellular events [33], infer physical properties [34], and reconstruct lineage trees [29]. In addition, cellular segmentation and tracking enable the unbiased inference of rules behind cellular behaviors using machine-learning techniques in homeostasis [35], and the methodology is readily adaptable to various developmental processes.

Since the growing size of a typical dataset has made purely manual analysis prohibitive, various automated computational methods have been developed to help the segmentation and tracking of single cells. Although non-machine-learning-based methods have been successfully applied to images with a high signal-to-noise ratio and clear features, these conditions are not always satisfied owing to fundamental constraints such as speed, resolution and phototoxicity [11]. More recently, machine-learning-based methods have been developed to yield better performance for challenging data with appropriate training datasets [3,4]. Moreover, there have been continuous efforts to implement those algorithms as publicly available software tools usable without expertise. As current machine-learning-based methods often require manual annotation and validation, various software programs have also been developed for this purpose.

In this section, we focus on instance segmentation and cell tracking techniques necessary for those analyses and introduce the algorithms and software developed thus far. In particular, from the perspective of reproducibility and shareability of the analysis, we emphasize open-source software accessible to non-experts. Many tools can analyze 2D images of *ex vivo* tissues or cultured cell lines, as well as 3D *in vivo* images. We also briefly discuss image preprocessing.

### Workflow for single-cell image analysis

**Fig. 2.**
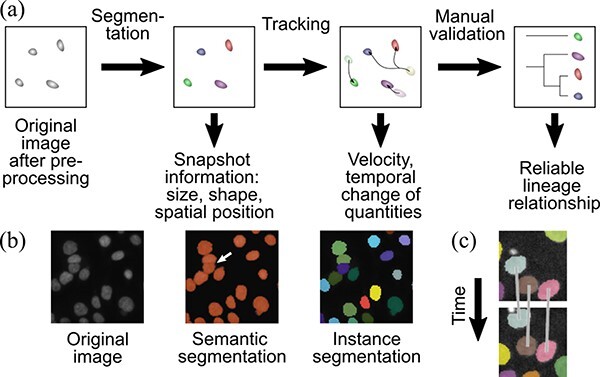
**Illustration of segmentation and tracking concepts** (a) Typical image processing workflow for single-cell scale segmentation and tracking analysis. The expected outcome is presented below each illustration. This workflow implicitly uses the tracking-by-detection approach (see main text). (b) Illustration of the semantic and instance segmentation. The colors represent the values obtained by the segmentation algorithms. In semantic segmentation, close objects are detected as a connected region (white arrow). The original image is taken from Ref. [188], licensed under the Creative Commons 0 license (See https://github.com/CellProfiler/examples/issues/41 for discussion). (c) Illustration of the tracking-by-detection approach. The segmented/detected cells are temporally connected to find tracks. The image is taken from Ref. [88], licensed under the Creative Commons Attribution license (https://creativecommons.org/licenses/by/4.0/).

Figure 2a illustrates the typical workflow of a single-cell scale analysis. In the workflow, the cells are detected and linked into tracks to construct cell lineages. In the following subsections, we review the algorithms and tools used for cell segmentation and tracking.

The required accuracy of the analysis and preferred labeling strategy vary depending on the purpose. For example, to quantify the population distribution of a snapshot without tracking, it may be sufficient to exclude failed segmentations according to some criteria. In contrast, to track cells over a long period with parent–child relationships, the segmentation and tracking should be highly accurate, given that the number of complete tracks exponentially decreases [36]. In particular, for 3D images and crowded cells, it is still challenging to reconstruct complete lineage trees over a long period [4,37] and manual curation often remains essential even with current state-of-the-art techniques [36,38]. Tracking sparsely labeled cells or regions instead of all cells can be less challenging and may be preferred when dense tracks are not required [29,39]. A notable example is the analysis method proposed in Ref. [40] that enables inference of deformation dynamics of 2D epithelial sheets in the 3D space using trajectories of sparsely labeled cells or markers attached on the tissue.

### Cell segmentation tools

To quantify images at the cellular level, it is necessary to detect the locations of cells or segment cellular regions from the background and adjacent cells. This subsection particularly focuses on segmentation methods [3,41–43] that can provide information on the morphologies as well as positions.

Semantic or instance segmentation methods are frequently used for segmenting cellular or nuclear areas [3] (Fig. 2b). Semantic segmentation divides a region into object classes by assigning a label to each pixel and does not distinguish individual objects connected to or overlap each other (Fig. 2b, center). In contrast, instance segmentation identifies each object and thus enables the quantification of individual cell properties (Fig. 2b, right). Note that semantic segmentation is sometimes performed as a processing step for the instance segmentation.

Classically, methods such as thresholding followed by watershed separation or edge detection have been used for the instance segmentation [41,42]. Software such as ImageJ [44] and scikit-image [45] implement various functions for these tasks. In addition, tools such as Jupyter Notebook (https://jupyter.org/) [46] and napari [47,48] can be used for interactive analysis in Python. MorphoGraphX [49,50] is a GUI software that enables the segmentation of cells on curved surfaces by the watershed method. TissueAnalyzer [51] (formerly known as the packing analyzer [52]) also implements the watershed method as well as downstream analysis functions. See Refs. [41,42] for a review of these methods and Refs. [53,54] for practical tutorials. These methods have the advantages of requiring fewer parameters to be adjusted, allowing segmentation even without annotated data, and often exhibiting computational performance superiority compared to machine-learning methods. For example, in Ref. [55], the authors developed a robust segmentation method for membrane-stained embryos, enabling the quantification of single-cell morphogenetic changes during developmental processes. However, obtaining accurate segmentation using these methods can be more challenging with low signal-to-noise ratio, non-uniform staining or complex cell morphology.

Machine-learning-based methods that exhibit robust performance have been actively developed and utilized when appropriate training datasets can be prepared. For example, those algorithms are outperforming the others for a majority of datasets in the Cell Tracking Challenge (CTC) [4], indicating that machine learning can be a better option when sufficient training data are available. In particular, deep neural network models, such as the U-Net [56], are a popular choice among segmentation algorithms [57], as can be seen with the CTC participants [4] and published algorithms comprehensively reviewed in Ref. [3]. While general network structures and models such as Mask R-CNN (Region-based Convolutional Neural Network) [58] have been used for cell segmentation [59], specific model architectures have also been proposed for better performance [3,60–64]. There has been a growing effort to make these models widely accessible as Python packages. Examples include StarDist [60], Cellpose [62,63] and EmbedSeg [64], some of which can also be used in ImageJ [44,65–67].

Despite their performance, a majority of current methods for cell segmentation are supervised machine-learning methods that require a large amount of manually validated training data, and laborious validation and annotation processes often hinder their application to new datasets. To overcome this problem, some software programs have adopted pretraining models with images from various modalities. For example, the BioImage Model Zoo [68] releases pretrained models based on multiple datasets that users can use in Python, ImageJ [44] (using deepImageJ [67]), ilastik [69], ImJoy [70], Icy [71] and QuPath [72]. Pretrained models have also been released for models such as Memster [73] (https://github.com/vanvalenlab/intro-to-deepcell), LIVECell [74] (https://github.com/sartorius-research/LIVECell), and Cellpose (https://github.com/MouseLand/cellpose) [62,63]. Weakly supervised methods that use abundant unlabeled images and labeled images can also be useful to avoid the bottleneck in the annotation. A competition for multi-modal weakly supervised learning models was recently held at the Thirty-sixth Conference on Neural Information Processing Systems (https://neurips22-cellseg.grand-challenge.org/), where various methods have been benchmarked.

When pretrained models do not provide sufficient accuracy or not publicly available for target modality, it is necessary to train them using newly annotated ground-truth data. Training such models requires a certain expertise, but the effort has been devoted to making this process more available. For example, a step-by-step Jupyter notebook has been released [75] (https://github.com/HenriquesLab/ZeroCostDL4Mic), allowing users to download models from the BioImage Model Zoo and retrain them on real data. A GUI for training has made the training process more accessible [63]. We will further review available software packages and strategies for efficient manual annotation later.

### Cell tracking tools

After the segmentation, tracking of the segmented regions is essential for understanding temporal property changes and lineage differentiation (Fig. 2a). Recently, there has been a shift towards automated tracking using various algorithms instead of the traditional labor-intensive manual process [37,43,76].

The cell tracking task can be framed within the broader context of computer vision, specifically as a multi-object tracking task [77,78]. Although various methods developed for general tasks can be used, several tools and algorithms have also been specifically tailored for cell tracking.

Several groups have published workflows of cell detection or segmentation and tracking dedicated to the 3D nucleus- or membrane-stained developing embryos [29,36,55,79,80], which may also work for images with a similar modality. In this subsection, we introduce cell tracking algorithms and software tools from a general perspective.

It is helpful to categorize the methods based on the processing workflow, frames used for tracking, and types of cost functions. The first criterion categorizes the methods based on the relationship between segmentation or detection and tracking tasks. It is popular to employ the *tracking-by-detection* approach, in which the algorithm detects objects in advance and determines their optimal assignments [77,81] (Fig. 2c). This approach has the advantage of simplicity and allows users to combine different segmentation and tracking techniques, depending on their image modality. There are several methods that do not fit into this category, such as a machine-learning method that train the model simultaneously for detection and tracking tasks [82], template matching, detection and tracking by the Gaussian mixture models [29,55,80], and simultaneous segmentation and tracking by registration (e.g. ASTEC method [83]) or deformable contour models [76]. Regarding the second criterion, tracking methods that use only past frames are called *online* methods, whereas *offline* methods can use all the frames, including future ones. In general, offline methods have a performance advantage [77], whereas online methods can be applied to real-time experiments. The final criterion is in the form of the cost function. In several tracking algorithms, the assignments are performed by maximizing a function called the posterior distribution of the assignments [77] or, equivalently, minimizing a cost function whose value depends on the assignments [37]. The forms of the cost functions can be either heuristically assumed or learned from annotated data.

Heuristic cost functions allow for tracking without a training step. The algorithm can be simplified further if it assumes that the association probability between cells depends only on their properties in two consecutive frames. For example, by defining the cost function as the square of the particle displacement, this algorithm can be regarded as the maximum likelihood method for particles undergoing Brownian motion [84] and was adapted to particles with splitting and merging processes [85]. Software such as TrackMate [86,87], LapTrack [88], CellProfiler [89], u-track [85] and Lineage Mapper [90] implement similar tracking algorithms. Temporal information can also be used for Bayesian modeling by defining a cost function that depends on multiple frames. For example, tools such as btrack [91], TrackMate [86,87] and u-track [85] can perform cell tracking using the Kalman filter, which is particularly useful when temporal memory is present in the cell motion. Ilastik [69] and SpotTracking plugin in Icy [71] (https://icy.bioimageanalysis.org/plugin/spot-tracking/), respectively, implement different probabilistic models aware of under-segmentation [92] and undetected particles [93].

Machine-learning methods can be a suitable option when ground-truth annotations are available. Some works employ learning the temporal displacements of segmented regions. For example, the tracking module in ELEPHANT [79] uses the U-Net trained for the optical flow, whose output is used to improve the nearest-neighbor linking. Linajea [36,94] uses the U-Net to predict the displacement to the center of the identical object at the preceding frame, which is used to extract cell lineage graphs from possible detection and linking candidates. EmbedTrack [95], which is trained to predict the offsets between the positions of pixels belonging to a segmented cell region and the center position of the identical cell at the previous frame, has shown competitive performance for several 2D CTC datasets. Alternatively, some works take an approach to train a model for association probabilities. For example, graph neural network-based methods [96] and methods that learn the probability of cell association using neural networks [97] have been proposed. Ref. [98] uses convolutional neural networks to assign cell-cycle states which are used at tracking. Still, machine-learning-based tracking algorithms are undergoing active development, and there is a need for comprehensive benchmarking to elucidate how the performance depends on the amount of training data and how they generalize for different modalities.

One barrier to the machine-learning approach is the difficulty in preparing the training data. Incremental training using sparse annotation is a straightforward method for addressing this issue, and several tools have been developed in this direction. For example, the detection and tracking models of ELEPHANT [79] and Linajea [36,94] can be trained using sparse annotations, which enables incremental model performance improvement with feasible time. Alternatively, the parameters of heuristic models can be tuned using ground-truth annotations. LapTrack [88] allows tuning of the form of the cost function and parameter values with sparse ground-truth annotations. TrackMate [86,87] also has a similar parameter-tuning capability.

### Datasets and benchmarking

Benchmarking on datasets with ground-truth annotations is useful for determining how well these methods meet the required performance criteria. For example, using quality-assured datasets, CTC [4,99] has been continuing to benchmark segmentation and tracking methods, and the Kaggle 2018 Data Science Bowl [100] has performed segmentation benchmarks. In addition, there exist various publicly available datasets [3,62,101] that can be used for benchmarking.

Although these benchmark results are useful for inferring the effectiveness of the method, it is important to note that they cannot be used as the sole basis for usability. This is because (i) different datasets have different annotation standards and qualities [3,63]; (ii) the benchmark ranks can be affected by the choice of annotators, test data and metrics [102] that do not necessarily correlate with biologically relevant performance [103]; and (iii) although benchmarks typically use a large number of annotated datasets for training, it is time-consuming to prepare a large number of annotations by experts from the beginning in real experiments. In practice, the optimal method will likely depend on the size of the human annotations, the microscopy technique, and the expertise of the researchers. Building a reproducible benchmarking platform [104], improving competition standards [102] and training sufficiently large and general models [105] can help improve this situation.

### Manual annotation tools and assistance through machine learning

Achieving high accuracy in the analysis, training supervised machine-learning models, and measuring the prediction performance often requires researchers to validate and correct segmentation and tracking results. Annotation tools play a crucial role in this context.

There are various manual segmentation annotation tools, as reviewed, for example, in Ref. [3]. In addition, napari [48] has recently been developed as a general image visualization software with the ability to edit segmentation masks for images of arbitrary dimensions [47]. Napari’s ecosystem enables users to extend the software using plugins for various tasks, including semantic and instance segmentation (https://www.napari-hub.org/). It can also be easily controlled using Python and integrated into a custom-built analysis workflow. In addition, Cellpose [62,63] is packaged with a GUI that supports editing segmentation for 2D images and *in situ* training and prediction of the instance segmentation model.

Although manual segmentation annotation for large images can be time-consuming, machine learning can assist this process. One example is the use of pixel classifiers, such as the Trainable Weka Segmentation in ImageJ [106,107], Labkit [108], ilastik [69], QuPath [72] and napari plugins [109,110]. By providing sparse manual annotation of different regions, these models can be trained for semantic segmentation by classifying each pixel based on its features, including the surrounding context. This method is particularly useful for objects that are not adjacent to each other or separated by clear boundaries because instance segmentation can be straightforwardly deduced from the result.

Pretraining can also be useful for generating annotated datasets with fewer manual annotations. As noted in previous subsections, there are several public segmentation models trained with various images. Even if the training dataset does not contain the same images as the target microscopy images, one can benefit from faster training than starting from scratch [63,70]. This can be interpreted in terms of *transfer learning* [111], which involves leveraging knowledge from a different domain to enhance the prediction performance.

Another important concept is the *human-in-the-loop* training [55,63,70,73,112], which encompasses an approach that iteratively performs annotation and training processes instead of annotating all data at once. The annotation of each iterations is used to train the model, and the trained model then generates a new segmentation that is again corrected by a human annotator. This approach can help decrease manual correction efforts to achieve similar performances [63,70].

Although annotation and correction of tracking results are more challenging than segmentation, several open-source software programs have been developed for this purpose. For example, TrackMate [86,87] implements TrackScheme (https://imagej.net/plugins/trackmate/views/trackscheme) to edit the tracking results. In addition, various software packages support manual correction of segmentation-annotated [73,113–118] or point-annotated [119–123] tracking results. CATMAID [123], originally developed for large-scale 3D datasets, was successfully extended to manually curate cellular lineages [80]. The idea of human-in-loop training can be utilized for tracking. For example, ELEPHANT [79] is a pioneering software package for human-in-loop training for tracking that focuses on 3D ellipsoidal object tracking instead of pixel-segmented objects. The nucleus detection and motion estimation models were trained using sparse annotations, which enabled the iterative training of the tracking model. Annotation-preserving tracking [88] is another simple option for human-in-loop tracking, in which users can retrack cells that are not validated.

### Image preprocessing

Although this section focuses primarily on segmentation and tracking, preprocessing also plays a critical role in data quality and should be carefully performed. Tools regarding spatial and temporal inhomogeneities, as well as image noise, are briefly discussed here. Readers can refer to Refs. [43,76] for further review.

Signal inhomogeneity that does not exist in the original specimen is called *shading* and is caused by various optical factors [124]. This can affect the segmentation quality and quantitative interpretation [125]. To suppress this effect, it is often desirable to *prospectively* estimate and compensate for the shading patterns using special images without objects or *retrospectively* using the sample images themselves. Algorithms such as BaSiC [126] and CIDRE [125] have been proposed for retrospective correction, yielding better performance than naive methods. For multi-positional images, it is necessary to perform this operation before stitching. Several open-source tools, including those in Refs. [127–130], can be used for stitching after shading correction.

Especially in low-illumination cases, Poisson noise due to the discreteness of photons and readout circuit noise can affect the downstream analysis. Methods ranging from spatial filters to machine learning can be used to reduce the impact of noise [131].

The imaging and analysis methods have supported the elucidation of morphogenesis processes driven by, ultimately, mechanical forces. However, to infer *causal* relationships between mechanical forces and morphogenesis processes, mechanical stimulation and manipulation methods are essential. In the following section, we turn our attention to those methods.

## Mechanical signal in morphogenesis: manipulation tools and recent studies

Recent technological advancements have now made it possible to observe even the formerly ‘invisible’ elements, such as the mechanical interactions that influence multicellular tissues. Various methods have been developed for image-based force measurements to estimate the tension and pressure applied to cells. For example, Bayesian inference of membrane tension and cell pressure and statistical inference of mechanical models and their parameters have been proposed [34,132].

To gain insights into the roles of mechanical signals, it is necessary to analyze mechanical force as one of the biological signals, with the relationship among cellular motion, genetic networks and protein interactions. Similar to the perturbation of genes, such as knockdown or over-expression, the perturbation of mechanical signals is required to elucidate the function of the mechanical force within this relationship. Recently, precise and quantitative manipulation of mechanical forces has become possible using new microscopy techniques. In this section, we introduce tools for measuring and manipulating mechanical forces, along with their applications in cutting-edge studies.

Numerous manipulation techniques and force measurement equipment have been reported. We introduce the tools for applying mechanical perturbations at the cellular level and their applications. In particular, we focus here on tools that can be combined with optical microscopy introduced above, enabling the integration of mechanical perturbations with imaging capabilities.

### Laser dissection

One of the simplest methods to perturb a mechanical signal is the laser dissection, which can disrupt the balance of the force by cutting biological objects such as plasma membranes with a focused pulsed laser. One application of this technique is the measurement of the cortical tension. For instance, in Ref. [133], the authors observed the retraction of cell junctions in *Xenopus* gastrula mesoderm explants after laser dissection and computed the relative cortical tension.

Although laser dissection is a simple and easy method, its invasiveness restricts its application to observing specimen following dissection. On the other hand, non-invasive precise manipulation of force is essential to dissect the mechanical signal, particularly to demonstrate the adequacy of force as a sufficient condition, such as the activation of the signal by artificial force. In the following, we introduce techniques for manipulating mechanical signals.

### Magnetic tweezers

**Fig. 3.**
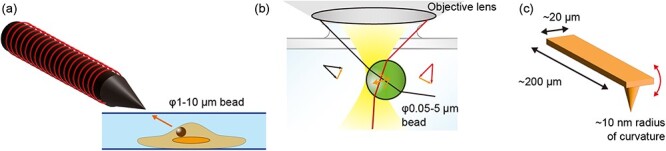
**Comparison of the manipulation techniques** (a) Schematic of a probe of magnetic tweezers. Typically, a solenoid (red line) wraps around a rod (black). The distance between the solenoid and the sample is commonly controlled by a manipulator (data not shown). A 1–10 µm-diameter magnetic particle is usually used for the probe (brown). (b) Schematic of optical tweezers. A laser beam is focused by an objective lens (illustrated as an ellipsoid), trapping a particle (green) with a high refractive index in the medium. The 0.05–5 µm diameter polystyrene particle is usually used for the probe (green). (c) Schematic of an AFM probe, using a fine probe that typically moves along the *z*-direction. AFM can manipulate the specific point in the sample. Generally, the specimen is fixed on the flat stage, such as the mica surface (data not shown), and the probe scans the surface.

Magnetic tweezers (Table 2, Fig. 3a) and techniques that employ an external magnetic field to manipulate magnetic particles [134,135] have been recognized as among the most effective tools for applying force to tissues. This technique applies forces ranging from approximately pico-Newtons to nano-Newtons to magnetic beads of a few micrometers in size. A solenoid wrapped around a rod is typically used as a probe for magnetic tweezers (Fig. 3a) [134,136]. There are also sophisticated magnetic tweezers that can apply torque [135,137] or generate strong local magnetic waves using Helmholtz coils and permalloys [138].

**Table 2.** Comparison of manipulation techniques

	Magnetic tweezers	Optical tweezers	AFM
Force range (pN)	0.01–10^4^	0.1–100	10–10^4^
Applications	Applicable to deep tissue Force clamp Bead rotation	3D manipulation Combinable with other microscopes (such as confocal microscopy)	High-resolution imaging Stiffness measurement
Limitations	Difficulty of fine manipulation	Photodamage (Due to use of high-power laser)	Limitation of specimen Relatively slow
Effective working distance	∼ Centimeter	<∼100 µm	<∼Angstrom
Applicational targets (in morphological studies)	Heart [139]	Cilia [14,153] Cell junction [151,152]	Blastocyst [158]

Given their relatively stronger force compared to the other techniques introduced below (Table 2), magnetic tweezers are suitable for multicellular systems. Fukui *et al*. demonstrated that ectopic shear stress induces Ca^2+^ activation by applying mechanical force to the zebrafish heart with magnetic tweezers. Further experiments revealed that Ca^2+^—NFAT (nuclear factor of activated T cells) signaling is activated in response to mechanical stimuli, which permissively controls valve morphogenesis in the heart [139].

Another interesting approach that uses magnetic fields is the use of magnetically responsive oil microdroplets [140]. When injected into living tissues, the droplets deform in response to an external magnetic field. The authors of Ref. [140] measured the yield stress at various points along the body axis, which represents mechanical integrity. By analyzing the shape change of the droplets, they found that a jamming transition from fluid to solid underlies the elongation of the body axis in vertebrates. Furthermore, the analysis of rheological responses revealed that presomitic mesoderm cells mechanically probe their microenvironments in zebrafish [141].

Although magnetic tweezers serve as an initial approach for tissue manipulation, it has a disadvantage in precisely controlling the displacement of the probe. By employing a positional feedback system to regulate the applied force, magnetic tweezers enable the positional manipulation of particles [142]. However, for precise positional manipulation, other tools, such as optical tweezers or atomic force microscopy (AFM), are more suitable. Those tools are detailed in the following.

### Optical tweezers

For accurate positional manipulation of probes at the nanometer level, optical tweezers are a good choice (Table 2, Fig. 3b). Optical tweezers trap a small particle using a focused laser beam [143]. When a particle with a high refractive index, such as a polystyrene bead, is placed in the path of the focused laser beam, the particle is trapped because of the force that originates from the slight refraction of the light (Fig. 3b). Since the technique requires the refractive index difference between the medium and the beads and laser focusing without optical aberrations, it is only applicable to the surface of the tissue, basically.

Typically, optical tweezers are constructed by introducing an infrared continuous wave laser beam into the microscope through the episcopic illuminator port and focusing the beam on the sample plane through a high-numerical-aperture objective lens [144]. This means that microscopes with an episcopic illuminator port can be equipped with optical tweezers, allowing seamless integration with other techniques.

Using high-accuracy actuators such as piezoelectric actuators, optical tweezers can manipulate a trapped particle with nanometer accuracy. Near the center of the beam, the force applied to the trapped particle increases linearly with the displacement from the beam center. This enables the determination of the force applied to the particle by the displacement. Indeed, high-precision measurement of the displacement of trapped particles can be achieved through the integration of a 3D single-particle tracking method [144,145] which enables the force measurement in 3D space with pico-Newtons accuracy [146].

Optical tweezers are suitable for single-molecule experiments owing to their high-positional accuracy, and thus, they are commonly combined with single-molecule imaging techniques [147,148]. This technique can also be used to measure the stall forces of bacteria [149] and organelles [14,146]. Furthermore, using a spatial light modulator, which generates multiple focused beams through the interference of a laser beam, optical tweezers can simultaneously trap multiple particles [150].

If the refractive index of the target is sufficiently larger than that of the medium, optical tweezers can be used to manipulate the target without beads. For example, cell junctions of the epithelial tissue in *Drosophila* embryos [151,152] or single cilium located in the left–right (L–R) organizer of zebrafish can be directly trapped by optical tweezers [153]. Nishizawa *et al*. succeeded in artificially inducing the remodeling of cell–cell junctions using two-point beadless optical tweezers and revealed how mechanical stresses lead to the efficient deformation of cell–cell contacts [151].

### Atomic force microscopy

Despite the limitation of specimens, AFM is useful for force manipulation and measurement combined with high-resolution imaging of surface topography (Table 2, Fig. 3c). AFM scans the height of the sample surface by detecting the force between the sharp-tip probe (typically with a tip radius of ∼10 nm) and the sample; therefore, the specimen should attach on the flat surface such as mica. AFM can also measure and apply forces in the range of pico-Newtons to nano-Newtons [154]. Various types of AFM have been reported [155], and particularly, recent development in high-speed AFM enables real-time measurement of live biological samples in a liquid environment with nanometer accuracy (typically with a spatial resolution of 2–3 nm and a time resolution of ∼100 ms) [156,157], as well as the simultaneous application of mechanical force to the desired point.

AFM can be utilized to characterize a multicellular system by, for example, measuring the blastocyst stiffness [158]. However, because of the requirement for samples to be tightly attached to mica or glass surfaces, AFM has been used less frequently in morphological studies. Nevertheless, combining AFM with optical microscopy is a promising approach for extending its applicability [154,159].

## Integration of imaging, analysis and mechanical manipulation toward understanding of morphogenesis processes

Integrations of the imaging techniques, image analysis tools and mechanical manipulation tools described so far are beginning to reveal the cellular and molecular mechanisms of various dynamic biological events, such as morphogenesis. Here, we overview how improvements in imaging speed and volume, advancement in analysis methods and cutting-edge force manipulation methods have enabled us to understand the orchestration of mechanical and molecular processes in development.

### Imaging-based studies revealed cellular and molecular mechanisms of collective deformation in embryogenesis

There are major morphogenesis processes that occur in relatively short timescales, and improvement in imaging speed has contributed to studies of those processes. Notable examples can be seen in the research of tissue invagination, such as gastrulation and neural tube formation [160,161]. Typically, tissue invagination is driven by apical constriction, a process characterized by the contraction of apical cell surfaces [162]. High-speed imaging using spinning-disk microscopy is essential to capture specific cellular and molecular dynamics in real-time, considering that cells change shape in a few minutes during apical constriction and that molecules driving apical constriction, such as actomyosin, can change their localization in tens of seconds (see Table1). For example, in *Drosophila* gastrulation, epithelial cell sheet folds as cells undergo pulsed apical contraction [163]. This correlates with the pulsate cellular motion that shows switching between contractile and stable phases every few minutes in a ratchet-like fashion. The ratchet-like apical constriction is driven by pulsed myosin coalescence, which changes the actomyosin structure within seconds [163]. Utilizing spinning-disk microscopy enabled high-speed imaging of the apical myosin dynamics. Recently, in mouse gastrulation, researchers have also shown that ratchet-like apical constriction drives tissue invagination during epithelial-to-mesenchymal transition (EMT), combining imaging by spinning-disk microscopy and automatic segmentation with Tissue Analyzer [52,164]. During EMT, cellular mechanisms other than apical constriction have also been reported to induce tissue folding. In *Drosophila*, integration of spinning-disk microscopy for rapid imaging of mesodermal invagination and automated segmentation revealed that ectopically induced EMT leads to cell delamination that exerts an apicobasal force on the epithelial sheet to fold tissue [165].

Convergent extension, such as body axis elongation, is another morphogenetic process that requires imaging speed due to its rapid progression. In convergent extension, cell intercalation, in which cells dynamically change their relative positions, is considered one of the driving forces [166]. In *Drosophila*, time-lapse observation revealed that the inter-cellular boundary perpendicular to the extension axis actively contracted and disappeared [167]. Subsequently, a boundary is newly formed along the extension axis, which rearranges the relative position of the four involved cells [167]. There is another type of cell intercalation involving five or more cells (up to about 10 cells) called ‘rosette’ formation [168]. During this process, cells all together shrink the cell membrane parallel to the axis of contraction, which converge the membrane vertices to a common point. Subsequently, new cell boundaries perpendicular to the axis of contraction are formed simultaneously in each cell, promoting tissue extension. Given the rapid cellular dynamics, spinning-disk microscopy was employed in these studies to acquire images at rapid intervals of several tens of seconds.

Deep tissue imaging using two-photon microscopy is also invaluable for studying morphogenetic mechanisms. Its effectiveness is illustrated by studies that underscore the role of basolateral protrusion besides apical deformation, which formerly received the main focus. As apical dynamics of cells are easily visualized, it is not surprising that apical deformation was considered an active driving force for morphogenesis until the 2000s. However, recently, two-photon imaging has been extending our understanding of the role of basolateral cellular dynamics. For example, during the germband extension in the *Drosophila* embryo, epithelial cells form a basolateral protrusion that involves cell intercalation and convergent extension [31]. In this study, using the combination of two- and multi-photon microscopy and automated segmentation with Packing Analyzer [52], Sun *et al*. revealed that basolateral protrusion occurs independently of apical constriction and that the coordination of these two processes promotes efficient rosette formation resulting in germband extension [31]. In *Drosophila* wing imaginal discs, tissue folding is driven by the basal expansion of epithelial cells through local degradation of the extracellular matrix and by apicobasal shortening through increased tension in the lateral membrane [169]. In this paper, a multiphoton microscope was used to image wing imaginal discs with a thickness of ∼50 µm for longer than 1 h with excellent signal-to-noise ratio.

New discoveries can also be made by combining different microscopy techniques. For example, John and Rauzi used spinning-disk microscopy and light-sheet microscopy to reveal that the coupling of apical constriction and cell intercalation causes simultaneous coordinated tissue folding and extension [170]. This combination was essential to analyze the correlation between entire tissue deformation and gene expression pattern obtained by a wide-field observation and the rapid changes of the membrane in each cell obtained by a fast timescale. Note that automatic segmentation using ASTEC [83] enabled the analysis of big imaging data obtained by multiple microscopy methods in this study [170]. In another example, by combining imaging by two-photon microscopy and light-sheet microscopy, membrane segmentation, and nuclear tracking of neuroepithelial cells, it was shown that mechano-chemical feedback of the Sonic hedgehog (SHH) signal is required for tissue protrusion in the forebrain of the chick embryo [171]. This combination of the microscopy techniques enabled quantification of multicellular dynamics in large, thick tissues such as ∼60 µm thick brains of embryos in culture. Also, by combining two-photon and spinning-disk microscopy with quantitative measurements of cellular properties using machine-learning-based segmentation by StarDist [60], Vignes *et al.* have recently shown that cell volume reduction induces tissue convergence during cardiovascular morphogenesis in zebrafish [172]. Spinning-disk microscopy was essential for the imaging analysis of the heart that beats over 100 bpm. This work also demonstrates how a robust, deep-learning-based detection system can compensate for the low signal-to-noise ratio in two-photon microscopy. These significant findings were achieved by synergistically combining state-of-the-art microscopy techniques and image-analysis toolkits.

### Recent cutting-edge study of mechanical signal: mechanical force and left–right determination

In this final section, we introduce one of the studies that combined imaging, analysis and manipulation techniques as an application of these techniques to illustrate how this combination is essential for revealing the role of mechanical force in morphological study. In this context, we present a study on the L–R axis determination during mouse embryo development [14]. The mechanism underlying the determination of L–R asymmetry has been a subject of controversy, specifically, it was not clear whether mechanical or chemical signals initiate the determination process. This study, using optical microscopic techniques such as optical tweezers, clearly demonstrates that a mechanical signal sufficiently initiates the L–R symmetry breaking.

**Fig. 4.**
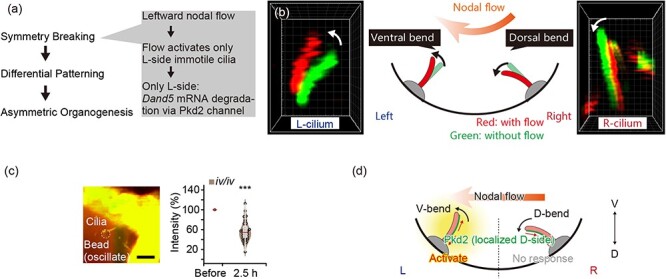
**Mechanical signals involved in the L–R determination** (a) Steps in the establishment of L–R asymmetry (left panel). The step of symmetry breaking includes three smaller steps (right gray box). (b) Measurement of the 3D deformation of the nodal immotile cilia through optical regulation of the nodal flow. The left and right panels show 3D reconstructed images of the left-side (L-side) and the right-side (R-side) cilium. Red and green colors represent the same cilium with and without nodal flow, respectively. The middle panel shows the schematic of the cross-section of the node. The L- and R-side cilia show ventral and dorsal bending by the nodal flow (illustrated in an orange arrow), respectively. (c) Manipulation of nodal immotile cilia by optical tweezers and measurement of the *Dand5* mRNA degradation. A polystyrene bead (white dotted circle) was trapped and oscillated along the *z*-axis by optical tweezers and contacted a cilium (left panel). *Dand5* mRNA degradation, the earliest marker for the L-side determination, was activated through mechanical stimuli of optical tweezers (right panel). To evaluate the response of the mechanical stimuli while excluding any influence from chemical cues, the authors used the *iv/iv* mutant, which lacks the nodal flow and flow-derived chemical cues. Even without chemical cues, mechanical signals activated mRNA degradation, which meant mechanical stimuli were sufficient to initiate the L–R determination. The authors measured the mRNA level by whole-cell fluorescence recovery after photobleaching [14]. The intensity was linearly correlated to the *Dand5* mRNA level. (d) Model of the initial L–R determination by the nodal immotile cilia. The cross-section of the node is shown. By the nodal flow (orange arrow), the L- and R-side cilia illustrated by the pink rods are bent to the ventral (V-bend) and dorsal (D-bend) sides, respectively. The Pkd2 channels are cation channels and one of the candidates of the mechanosensor on the cilia are localized on the dorsal side of both side cilium. On the L-side cilia, the membrane tension of the dorsal side is increased, which activates the dorsally localized Pkd2 channel. The *Dand5* mRNA degradation occurs only on the L-side and determines the L–R axis. (a–d) Modified from Ref. [14].

Why is the heart located on the left side of the body? During embryogenesis, a symmetric fertilized egg becomes an asymmetric body characterized by dorsoventral, anteroposterior, and L–R axes. Mechanical signals play an essential role in breaking L–R symmetry [14,153]. During mouse development, the L–R symmetry is first broken at the ventral node (the L–R organizer in this species) on embryonic day 7.5. The node has a leftward extracellular fluid flow known as the nodal flow [173,174]. Notably, the direction of this nodal flow determines L–R asymmetry through the primary cilia located at the peripheral region of the node, known as nodal immotile cilia [175–177] (Fig. 4a and b). Recent studies have revealed that nodal immotile cilia respond to nodal flow through the cation channel Pkd2 and activate the left-side specific signal, for example, the degradation of *Dand5* mRNA [176,178].

Most recently, state-of-the-art microscopic techniques have revealed that nodal immotile cilia sense the bending direction to sense the direction of nodal flow as mechanosensors [14]. In the study, Katoh *et al.* first found the passive mechanical motion of nodal immotile cilia by regulating the nodal flow by ultra-violet (UV) light. Nodal flow is generated by axonemal dynein [174,179], and UV irradiation induces the cleavage of the dynein heavy chain [180]. The authors constructed a microscope that was equipped with a spinning-disk confocal microscope and a UV-irradiation optical pathway, and stopped the nodal flow by irradiating strong UV light to the center region of the node. By obtaining 3D high-resolution images and comparing the angle change of nodal immotile cilia with and without nodal flow, it was found that nodal immotile cilia were passively bent by the nodal flow [14] (Fig. 4b).

Using these images, the authors then conducted a rigorous calculation to determine the strain in the ciliary membrane induced by nodal flow. The ciliary membrane of the images was modeled into approximately 2000 triangular mesh elements. The deformation of each element was measured by comparing the images with and without flow. Subsequently, the membrane tension of the cilium was calculated [181]. Their analysis revealed the membrane tension on the dorsal side of the left cilia is significantly increased by the nodal flow [14].

At the time of the study, it had been controversial how nodal immotile cilia activate. To directly test whether nodal immotile cilia can sense mechanical force, the authors directly applied mechanical force to a single nodal immotile cilium using optical tweezers. Cilia showed calcium transients in response to mechanical bending. Furthermore, 1.5 h of mechanical stimulation (left panel in Fig. 4c) activated *Dand5* mRNA degradation in the stimulated cells (right panel in Fig. 4c) and increased Nodal activity in the stimulated side of the node. These results suggest that mechanical stimuli to nodal immotile cilia are sufficient to initiate L–R symmetry breaking [14].

Experiments using optical tweezers demonstrated that the nodal immotile cilia act as mechanosensors. However, one important question still remained: Why are only the left side cilia activated by the leftward nodal flow? The authors provided insights into answering this question by demonstrating that nodal immotile cilia could only sense the ventral bend and did not respond to the dorsal bend. Super-resolution imaging with stimulated emission depletion (STED) microscopy revealed that the distribution of the cation channel, Pkd2, involved in L–R determination, was significantly biased toward the dorsal side of the nodal immotile cilia. Considering the finding that dorsal-side membrane tension was increased by nodal flow in the left-side cilia, it is likely that the dorsal membrane tension by flow will activate the dorsally localized Pkd2 channels only on the left side, but not in the dorsally bent right-side cilia. Indeed, the authors found that the cilia recognized the bending direction by 3D manipulation with optical tweezers. These results indicate that because the cilia can only sense the ventral bend, the nodal flow activates cells located only on the left side of the node, which leads to subsequent signaling cascades specific to the left side, such as Nodal signaling (Fig. 4d) [14].

## Conclusion and perspectives

In this review, we discussed recent advances in microscopy and image analysis techniques that can be applied to morphogenesis research. We also provided an overview of mechanobiological characterization and manipulation tools. Highlighting the findings of multicellular morphogenesis and mechano-signal transduction, our review illustrates how novel methodologies can advance our understanding in the fields of development and mechanobiology.

While we mainly focused on microscopy methods for live imaging, it is notable that recent technical developments have also enabled us to obtain highly multiplexed data from fixed samples. For example, spatial transcriptome technology has been used to investigate comprehensive gene expression patterns with positional information [182,183]. Additionally, highly multiplexed immunostaining can now be achieved through multi-round acquisition [184]. Analyzing those high-dimensional snapshot data and integrating them with timelapse images will likely provide unique opportunities for understanding regulatory mechanisms in developmental processes.

In this review, our focus has been primarily on supervised machine-learning methods. The annotation and validation processes can be a bottleneck of the training those supervised models, especially with an ever-increasing amount of image data. This situation highlights the importance of self-supervised and weakly supervised learning methods, which can utilize abundant non-labeled images, as emphasized in a recent review [185].

Despite the advancement of analysis algorithms and software tools, there is still room for user interface development and organizing documentation to ensure accessibility for non-specialists. This will necessitate further collaboration between biologists and computational experts, which may require reconsideration of data deposition methods and reward structures for scientists [186]. The application of large language models is a promising direction that may enable seamless communication between biologists, specialized analysis tools and even microscopes using natural languages [187].

While recent developments in imaging techniques have made it possible to describe detailed cellular dynamics at the organ scale, mechanobiology at the cellular and multicellular scale remains a budding research area that requires improvements in force measurement and manipulation techniques. As image acquisition capabilities increase, concurrent advances in force measurement and manipulation techniques are also needed. Although deep tissue imaging capabilities have expanded, 3D force estimation of cells is still challenging, which is necessary to unveil the mechanical signal underlying multi-cellular systems. Furthermore, although wide-field imaging through light-sheet microscopy is now possible, force manipulation is still limited to a range of a few hundred micrometers at most. Advances in microscopy and image analysis techniques can synergize with the development of force quantification and manipulation tools, which will uncover the mechanical aspects of morphogenesis.
